# Synthetic data for pharmacogenetics: enabling scalable and secure research

**DOI:** 10.1093/jamiaopen/ooaf107

**Published:** 2025-10-03

**Authors:** Marko Miletic, Anna Bollinger, Samuel S Allemann, Murat Sariyar

**Affiliations:** Institute for Optimisation and Data Analysis (IODA), Bern University of Applied Sciences, Biel, Switzerland; Department of Pharmaceutical Sciences, University of Basel, Basel, Switzerland; Department of Pharmaceutical Sciences, University of Basel, Basel, Switzerland; Institute for Optimisation and Data Analysis (IODA), Bern University of Applied Sciences, Biel, Switzerland

**Keywords:** artificial intelligence in healthcare, pharmacogenetics, genomic data, synthetic data, data privacy

## Abstract

**Objective:**

This study evaluates the performance of 7 synthetic data generation (SDG) methods—synthpop, avatar, copula, copulagan, ctgan, tvae, and the large language models-based tabula—for supporting pharmacogenetics (PGx) research.

**Materials and Methods:**

We used PGx profiles from 142 patients with adverse drug reactions or therapeutic failures, considering 2 scenarios: (1) a high-dimensional genotype dataset (104 variables) and (2) a phenotype dataset (24 variables). Models were assessed for (1) broad utility using propensity score mean squared error (pMSE), (2) specific utility via weighted $F_{1}$ score in a Train-Synthetic-Test-Real framework, and (3) privacy risk as ε-identifiability.

**Results:**

Copula and synthpop consistently achieved strong performance across both datasets, combining low ε-identifiability (0.25-0.35) with competitive utility. Deep learning models like tabula and tvae trained for 10 000 epochs achieved lower pMSE but had higher ε-identifiability (>0.4) and limited gains in predictive performance. Specific utility was only weakly correlated with broad utility, indicating that distributional fidelity does not ensure predictive relevance. Copula and synthpop often outperformed original data in weighted $F_{1}$ scores, especially under noise or data imbalance.

**Discussion:**

While deep learning models can achieve high distributional fidelity (pMSE), they often incur elevated ε-identifiability, raising privacy concerns. Traditional methods like copula and synthpop consistently offer robust utility and lower re-identification risk, particularly for high-dimensional data. Importantly, general utility does not predict specific utility ($F_{1}$ score), emphasizing the need for multimetric evaluation.

**Conclusion:**

No single SDG method dominated across all criteria. For privacy-sensitive PGx applications, classical methods such as copula and synthpop offer a reliable trade-off between utility and privacy, making them preferable for high-dimensional, limited-sample settings.

## Background and significance

Pharmacogenetics examines how an individual’s genetic makeup affects their response to medications.^1^ By understanding genetic variations that affect drug metabolism, PGx enables the development of personalized treatment plans that optimize therapeutic outcomes, eg, maximize efficacy and minimize adverse effects.^2^ For example, in the case of clopidogrel, genetic testing can identify patients with reduced CYP2C19 enzyme activity, which impairs the conversion of clopidogrel into its active form.^3^ This can lead to inadequate platelet inhibition, increasing the risk of cardiovascular events such as heart attacks and strokes. In such cases, alternative therapies or adjusted dosages are recommended to prevent these adverse drug events.^4^

Several challenges impede the full implementation of PGx in personalized medicine. A key issue is data scarcity, as PGx research requires large, diverse datasets to identify genetic variants linked to drug responses across different populations.^5^ The availability of such data is constrained by the high costs and complexities of large-scale genomic studies. Additionally, privacy concerns further complicate data sharing and access.^6^ Clinical PGx data is highly sensitive, usually encompassing both genetic and medical information, which increases the risks of re-identification and misuse if not adequately protected. Strict regulations and ethical guidelines governing patient data access create significant barriers to research, limiting the availability of crucial information. Collectively, these challenges slow the development and clinical application of PGx findings, restricting their broader impact on personalized medicine.

Synthetic data promise to address the key limitations of PGx research. Generated through advanced algorithms like generative adversarial networks (GANs) or variational autoencoders, synthetic data can mimic real patient data while preserving privacy by eliminating direct ties to individuals.^7^ This approach allows researchers to generate diverse, large-scale datasets that are representative of broader populations, improving model generalizability and reducing biases associated with underrepresented groups.^8^ Moreover, synthetic datasets can be tailored to specific research needs, enabling simulation studies and hypothesis testing that would otherwise be infeasible.^9^ It is often unclear whether synthetic data can truly replace original data in such a way that similar or even better results are achieved.

Previous applications of synthetic data in healthcare have demonstrated its potential across multiple domains, including research advancement and patient privacy protection.^10^ Various methods for generating synthetic genomes have been developed, utilizing diverse sources of information such as haplotype data,^11^ demographic details, and recombination patterns.^12^ More sophisticated approaches, including deep learning techniques like GANs and restricted Boltzmann machines (RBMs), have successfully produced synthetic genomic data that preserves population structure and variant frequency features.^7^ A recent study by Oprisanu et al evaluated the utility and privacy of these synthetic data generation (SDG) methods, indicating that recombination-based approaches offer high utility but low privacy, while RBMs present a balanced trade-off.^13^ In general, generating data distributions that closely mirror real data can make individual data points susceptible to membership inference attacks.^14^

The dependency structure among individual genetic markers is often highly intricate, making the use of advanced SDG techniques particularly promising for addressing key challenges such as missing data and class imbalances.^15^ This is not a new development, as synthetic data have been produced for genetic research for several years.^16^^,^^17^ What is novel, however, is the application of deep learning techniques capable of learning entire data distributions so effectively that they can even yield superior results compared to original datasets. Nonetheless, maintaining all statistical properties of real genomic data remains challenging, despite the synthetic data statistically mirroring real data. Furthermore, this approach is susceptible to membership inference,^18^ Additionally, synthetic data, although highly realistic, can still introduce biases and errors, such as mode collapse or distributional shift.^19^

In the field of PGx, the adoption of SDG techniques has lagged significantly behind other research domains, such as oncology.^20^ There is one notable exception, a study investigating a single gene, CYP3A5 (cytochrome P450 enzyme 3A5), utilizing a restricted set of variables.^8^ While this study demonstrates the feasibility of synthetic data for specific use cases, its scope is insufficient to derive generalizable insights into the efficacy and applicability of various SDG methodologies for broader PGx research.

## Objective

The objective of this article is to assess the potential of synthetic data in advancing PGx research by evaluating the following SDG methodologies: synthpop,^21^ avatar,^8^^,^^22^ copula,^23^ copulagan,^24^ ctgan,^25^ tvae,^25^ and tabula for large language models (LLMs),^26^^,^^27^ the latter of which leverages distilled GPT-2 by default. Our primary focus lies in analyzing the effectiveness of these techniques in high-dimensional settings that mirror the complexity of PGx datasets. Through this comprehensive evaluation, we aim to provide actionable insights for PGx researchers and practitioners, offering evidence-based recommendations for selecting the most suitable SDG methods. By bridging the current methodological gap, this study seeks to enhance the integration of synthetic data into PGx workflows, thereby facilitating innovation in personalized medicine.

## Materials and methods

### Real-world data basis: a Swiss PGx cohort

The dataset used in this study originates from a PGx case series conducted between 2019 and 2021 at primary and secondary care centers in Switzerland (ClinicalTrials.gov ID: NCT04154553).^28^ The study population included 142 adult patients referred due to adverse drug reactions (ADR) or therapy failures from a drug therapy, suspecting a potential drug-gene interactions (DGI). Patients underwent PGx testing using a commercial panel covering over 100 genetic variants across 30 genes, that are involved in the pharmacokinetic and pharmacodynamic processes of PGx relevant drugs. Collected data encompassed patient demographics, clinical diagnoses coded according to the ICD-10 classification, number of prescribed medications, suspected medication for DGI, and PGx testing results. Results of PGx testing are available as genotypes and genotype-predicted phenotypes. Following PGx analysis, a structured medication review yielded personalized therapeutic recommendations for patients and their physicians. Six months later, follow-up interviews assessed medication changes. The dataset represents a diverse clinical population, primarily with mental and behavioral disorders (61%), musculoskeletal, and circulatory system conditions.

To generate synthetic data, we consider the 2 detail levels: (1) High-dimensional genotypic data, which involves a dataset at the granularity of individual genetic variants per gene. For example, variants such as rs10509681 in CYP2C8 (A > G) and rs11572080 in CYP2C8 (G > A) are included, resulting in a total of 104 variables. This dataset is referred to as the “genotype” dataset. (2) Phenotypic data covering containing phenotype information without direct inclusion of genetic variants. This dataset is referred to as the “phenotype” dataset and consists of 24 columns.

In the preprocessing stage, minimal alterations were made to preserve the integrity of the dataset. The “changedDrug” column was truncated to generalize each drug entry from the chemical substance level to the pharmacological subgroup level, following the Anatomical Therapeutic Chemical classification (ATC) system. Additionally, identifying columns such as “id” were removed to ensure anonymization. No handling of missing values or outliers was necessary, as the dataset was already cleansed when received.

### Synthetic data generation methods

We employ a range of traditional, statistically grounded SDG methods. Synthpop models conditional feature distributions using techniques such as CART, effectively handling mixed data types. Copula-based models generate multivariate data by combining marginal distributions with dependency structures, primarily for continuous variables, with categorical data accommodated via transformations (eg, one-hot or ordinal encoding). The avatar method synthesizes data by resampling in a PCA-reduced latent space: data are standardized, embedded via PCA, and each point’s *k* + 1 nearest neighbors are identified. Synthetic samples are generated through inverse-distance-weighted combinations of neighbor embeddings, with added exponential noise to preserve local structure and enhance variability. Avatar’s integration of PCA and KNN enables efficient synthesis of high-dimensional, heterogeneous data.

As deep learning based generative models, we employ copulagan, ctgan, tabular variational autoencoder (tvae), and the LLM-based tabula. Copulagan integrates copula-based marginal modeling with ctgan to better handle mixed data types. Ctgan uses conditional GANs with mode-specific normalization to model imbalanced and high-cardinality categorical data. Tvae applies variational autoencoders to learn nonlinear dependencies via latent representations. Tabula treats tabular data as sequences, training a transformer-based language model from scratch to generate data via next-token prediction. Efficiency is enhanced through sequence compression and optional middle padding. While the distilled GPT-2 model used in Tabula does not meet the ≥1B parameter LLM threshold, we refer to it as LLM-based for simplicity.

We implement copula, copulagan, ctgan, and tvae using the Synthetic Data Vault (SDV) library.^29^ Synthpop is run in its native R environment for stability and full functionality. Avatar is reimplemented in Python, adapted from the simplified R version by Woillard et al,^8^ based on the original specification by Guillaudeux et al.^22^ For Tabula, we build on the open-source Python implementation by Zhao et al.^27^

### Utility and privacy measures

To evaluate the quality of the synthetic data, we employ 3 key metrics. First, we employ the Train-Synthetic-Test-Real approach, where models are trained on synthetic data and evaluated on original data, using the weighted $F_{1}$ score as the primary measure (1). Specifically, the original dataset is split into 80% training and 20% testing subsets. The SDG methods are trained on the 80% training split, and an equivalent amount of synthetic data is generated. Models trained on this synthetic data are then evaluated against the original 20% testing set. The weighted $F_{1}$ score obtained from models trained and tested on the original data is included as a reference, shown as a dashed line in the result visualizations.


(1) $$F_{1}^{w}=\sum_{i=1}^{C}\frac{n_{i}}{N}\cdot F_{1_{i}}$$

Second, we calculate the propensity score mean squared error (pMSE), as given in (2). Computed based on a CART classifier, pMSE is particularly suitable for this context, as it frames the problem as a classification task where poor classification performance is ideal—meaning synthetic records are indistinguishable from real ones. Lower pMSE values indicate better utility.^30^ When generating the same number of synthetic records as in the original data, the maximum pMSE value is 0.25; we normalize this value to rescale the metric between 0 and 1.


(2) $$\frac{1}{n_{1}+n_{2}}\sum_{n=1}^{N}{\left({\hat{p}}_{i}-\frac{n_{2}}{n_{1}+n_{2}}\right)}^{2}$$

Third, we assess the re-identification risk of synthetic data using the concept of ε-identifiability (3).^31^ This metric assesses the probability that a given record, denoted as in the original dataset has a smaller (weighted) distance to its nearest synthetic counterpart ${\hat{r}}_{i}$ than to its nearest original observation, $r_{i}$ where *ε* represents the threshold for closeness. The underlying principle is that lower uncertainty results in higher identifiability. In summary, a synthetic dataset $\hat{D}$ is ε-identifiable from the original dataset D if the following condition holds (δ is the indicator function):


(3) $$I(D,\hat{D})=\frac{1}{N}\sum_{i=1}^{N}\delta ({\hat{r}}_{i}<r_{i})<\varepsilon$$

### Evaluation design

We perform 100 independent sampling iterations for each SDG method and each fixed hyperparameter combination to robustly assess stochastic variability due to the inherent randomness of the algorithms. This procedure ensures that our analysis separates algorithmic robustness from the influence of hyperparameter tuning. Detailed results, including boxplots showing the variability across runs, are presented in Supplementary Material S3.

For deep learning-based methods, we evaluate 3 training epochs (300, 1000, and 10 000) on both genotype and phenotype datasets, using a constant batch size of 500. For LLMs, the batch size is consistently set to 64. Thus, across both datasets, training epochs vary systematically, while batch sizes are held constant to enable meaningful comparison.^32^

For Avatar, which does not depend on training epochs or batch sizes, we systematically explore key hyperparameters by varying the number of nearest neighbors (*k*) and the dimensionality reduction settings (PCA) across both datasets, following established practices in the literature.^8^^,^^22^ Specifically, we evaluate *k* = 5, 10, 15 combined with 45 and 104 PCA components for the genotype dataset, and with 20 and 24 PCA components for the phenotype dataset. Notably, the lower number of PCA components were chosen to capture 95% of variability in the data, ensuring that most of the original variance is retained even with reduced dimensionality.

**Figure 1.**
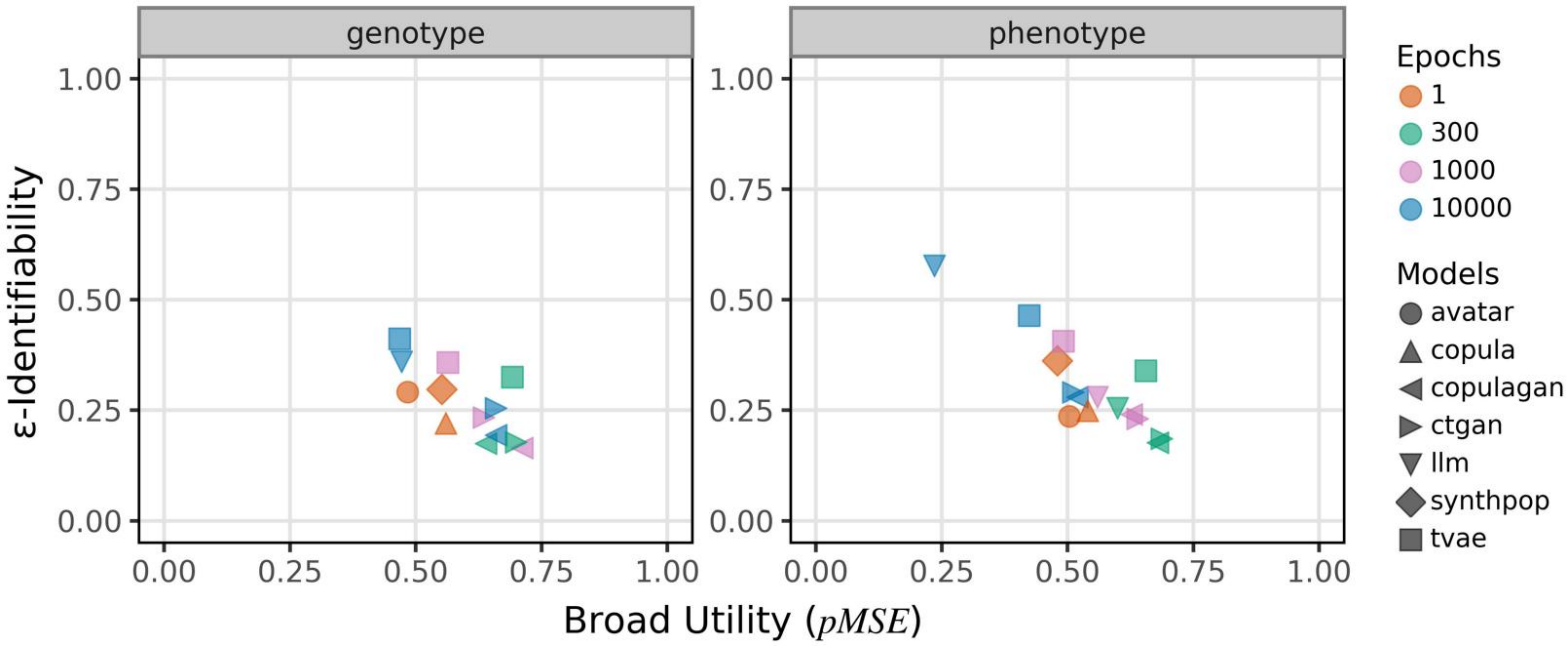
Relationship between broad utility (pMSE) and re-identification risk (ε-identifiability) for synthetic datasets generated by 7 models on genotype (left) and phenotype (right) datasets. Each marker represents a specific model-epoch combination, with shapes denoting models (avatar, copula, copulagan, ctgan, LLM, synthpop, tvae) and colors indicating training duration (1, 300, 1000, or 10 000 epochs). Axes are shared across panels to facilitate direct comparison. Lower ε-identifiability reflects smaller re-identification risk, while lower pMSE corresponds to greater broad utility.

**Figure 2.**
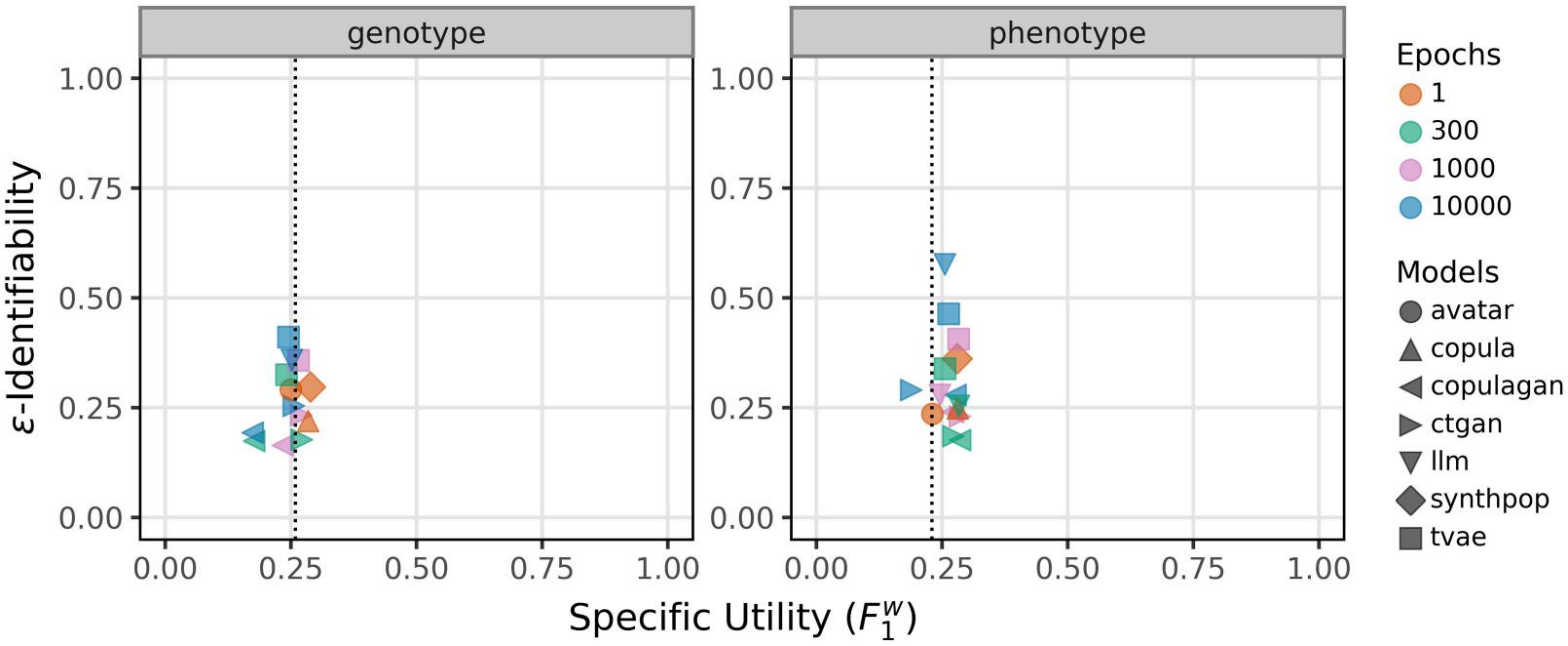
Relationship between specific utility ($F_{1}^{w}$) and re-identification risk (ε-identifiability) for synthetic datasets generated by 7 models on genotype (left) and phenotype (right) datasets. Each marker represents a specific model-epoch combination, with shapes denoting models and colors indicating training duration (1, 300, 1000, or 10 000 epochs). Dotted vertical lines indicate baseline $F_{1}^{w}$ scores (genotype: 0.259; phenotype: 0.230). Lower ε-identifiability reflects smaller re-identification risk, while higher $F_{1}^{w}$scores correspond to higher specific utility.

**Figure 3.**
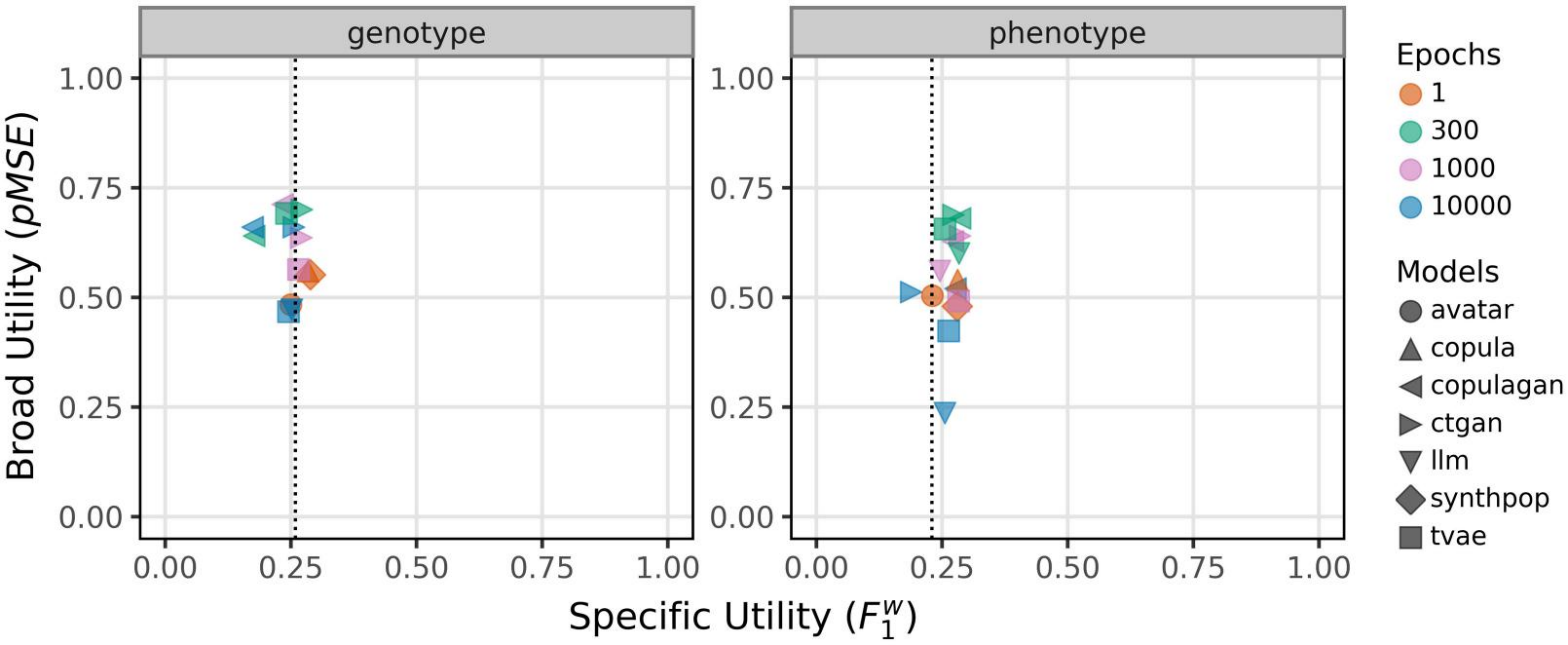
Relationship between specific utility ($F_{1}^{w}$) and broad utility (pMSE) for synthetic datasets generated by 7 models on genotype (left) and phenotype (right) datasets. Each marker represents a specific model-epoch combination, with shapes denoting models and colors indicating training duration (1, 300, 1000, or 10 000 epochs). Dotted vertical lines indicate baseline $F_{1}^{w}$ scores (genotype: 0.259; phenotype: 0.230). Lower pMSE values indicate greater broad utility, while higher $F_{1}^{w}$scores reflect higher specific utility.

However, integrating these variants into the same plots used for epoch-based deep learning models would conflate fundamentally different parameter types and lead to misleading comparisons. To enable a clear and fair side-by-side evaluation in the main manuscript figures (Figures 1-3), we therefore report results for a single representative Avatar configuration. This configuration (*k* = 10 with no dimensionality reduction) was selected through internal evaluation as the best trade-off between privacy and utility, based on the geometric mean computation of the metrics described below.

For synthpop, we use CART except for the first variable which is sampled due to having no prior predictors. CART hyperparameters are set to default values. The predictor matrix is left unmodified, ie, earlier variables are not excluded as predictors for later ones. All these hyperparameter choices reflect a calibrated privacy-utility trade-off and ensure comparability across methods by harmonizing key optimization parameters such as batch size and epoch count.

We compute the utility ($F_{1}^{w},\;\mathrm{pMSE})$ and privacy metrics (ε-identifiability) for each SDG method and hyper-parameter configuration and report their mean value across the 100 samples. Gini and permutation-based variable importance (VIMP) scores are presented for the best- and worst-performing methods, as determined by the geometric mean of the 3 metrics, using the inverse of $F_{1}^{w}$ to ensure a consistent interpretation direction across all metrics, where higher values indicate poorer performance.

The evaluation experiments were conducted on a server running Ubuntu 24.04 LTS and utilizing Cuda Toolkit 12.4 drivers. The hardware setup included 2 NVIDIA H100 PCIe 80GB graphics cards, 2 Intel Xeon Silver 4416+ processors, and 1 TB of RAM.

## Results

Figure 1 illustrates how SDG methods balance ε-identifiability (privacy risk) against broad utility (pMSE) across genotype and phenotype datasets. Traditional methods that do not involve epoch-based iterative training (synthpop, copula, avatar) are shown in red (epoch = 1), while deep learning-based methods are trained for 300, 1000, or 10 000 epochs (green, violet, and blue points).

In the high-dimensional genotype dataset, traditional generative models generally yield low ε-identifiability and moderate to good utility (pMSE between 0.45 and 0.6), indicating that non-deep-learning approaches can achieve a favorable privacy-utility trade-off even in complex data settings. Among these, copula and avatar perform particularly well, offering a balanced compromise between re-identification risk and data fidelity. In contrast, deep learning models such as LLM and tvae, especially when trained for 10 000 epochs, exhibit increased ε-identifiability with only marginal utility gains—suggesting overfitting and heightened privacy leakage. Notably, for LLM, data generation was only feasible at 10 000 epochs due to the limited dataset size, which hindered the model’s ability to learn the underlying distribution effectively at lower epoch counts.

In the lower-dimensional phenotype dataset, similar patterns are observed but with a more pronounced separation between methods in terms of ε-identifiability and pMSE values. The privacy-utility trade-off becomes more distinct, as methods like LLM at 10 000 epochs achieve significantly lower pMSE values, but only at the cost of substantially increased ε-identifiability—raising potential privacy concerns. Here, the strengths of copula and avatar become more evident: while they may not match LLM’s utility (a pMSE difference of ∼0.25), their significantly lower ε-identifiability justifies their use in privacy-sensitive contexts such as PGx. In other words, these traditional methods continue to offer a robust compromise, maintaining stable privacy while delivering reasonable utility, underscoring their practical relevance for SDG in real-world biomedical applications.

Figure 2 replicates the privacy-utility analysis, substituting the broad utility metric (pMSE) with the $F_{1}^{w}$ score to assess specific utility. In the genotype panel (left), most models cluster around $F_{1}^{w}$ scores close to the baseline (indicated by the dotted vertical line), suggesting modest performance on the downstream classification task similar to the original data. Privacy-wise, ε-identifiability remains moderate (0.25-0.5) for most models, with no strong correlation between training duration and increased privacy risk. Notably, copulagan and ctgan (at 300-1000 epochs) achieve relatively low ε-identifiability while maintaining $F_{1}^{w}$ scores on par with or above other models. Traditional methods perform less favorably, offering a weaker privacy-utility trade-off compared to the pMSE comparison. Meanwhile, the LLM model trained for 10 000 epochs exhibits markedly elevated ε-identifiability without a corresponding improvement in classification performance, suggesting overfitting or memorization rather than meaningful generalization.

In the phenotype panel (right), a comparable trend is observed: most models achieve $F_{1}^{w}$ scores close to or slightly above the baseline. Notably, ctgan and copulagan outperform other methods, which challenges the conventional assumption that deep learning models are better suited for high-dimensional data, while traditional methods excel in low-dimensional settings. Furthermore, our results underscore that evaluation metrics may behave counterintuitively and are not necessarily consistent with one another. For example, as demonstrated in Supplementary Material S1, models with superior pMSE performance often exhibit larger discrepancies in linkage disequilibrium (LD) structure compared to lower-performing models. Similarly, univariate distribution comparisons in Supplementary Material S2 reveal that strong alignment in marginal distributions does not reliably translate to improvements in other metrics. These findings emphasize that metric-specific optimization may obscure important deficiencies in the preservation of complex multivariate relationships.

Figure 3 illustrates the relationship between broad utility (pMSE) and specific utility ($F_{1}^{w}$). Across both genotype and phenotype datasets, models exhibit greater variability along the pMSE axis than along the $F_{1}^{w}$ axis. Most models cluster near the baseline $F_{1}^{w}$ score, indicating modest predictive utility when classifiers are trained on synthetic data. However, copula and synthpop stand out in the genotype dataset, where they solely exceed the baseline, suggesting that carefully designed statistical models can preserve task-relevant relationships more effectively than deep generative models. Conversely, methods such as LLM (10 000 epochs) and avatar highlight that low pMSE does not guarantee high task-specific performance. These results emphasize the critical distinction between fidelity (distributional similarity) and functional performance (predictive utility). Optimizing for one does not ensure improvement in the other—underscoring the necessity of a dual-metric evaluation framework when assessing synthetic data quality in privacy-sensitive domains such as PGx.

In the phenotype panel (right), models exhibit greater dispersion across the pMSE metric. The baseline $F_{1}^{w}$ is lower in this setting, reflecting the simpler and more structured nature of the phenotype data. Here, the LLM model trained for 10 000 epochs stands out by achieving the lowest pMSE among all models—suggesting high alignment with the original data distribution—while maintaining an average $F_{1}^{w}$ score. This combination yields the most favorable trade-off between broad and specific utility in the phenotype setting, despite the model not improving predictive performance beyond the baseline.

To further characterize method robustness, we include boxplots of metric variability in the Supplementary Material S1. These plots reveal that synthpop exhibits the highest stability across utility and privacy measures, while avatar displays substantial variability. Among deep learning-based models, variability is moderate overall, although tvae occasionally demonstrates greater instability, particularly in pMSE. Given that both tvae and avatar operate in latent spaces, one might cautiously hypothesize that latent-space-based methods are more susceptible to variability due to sensitivity in how they model and reconstruct the data manifold.

**Figure 4.**
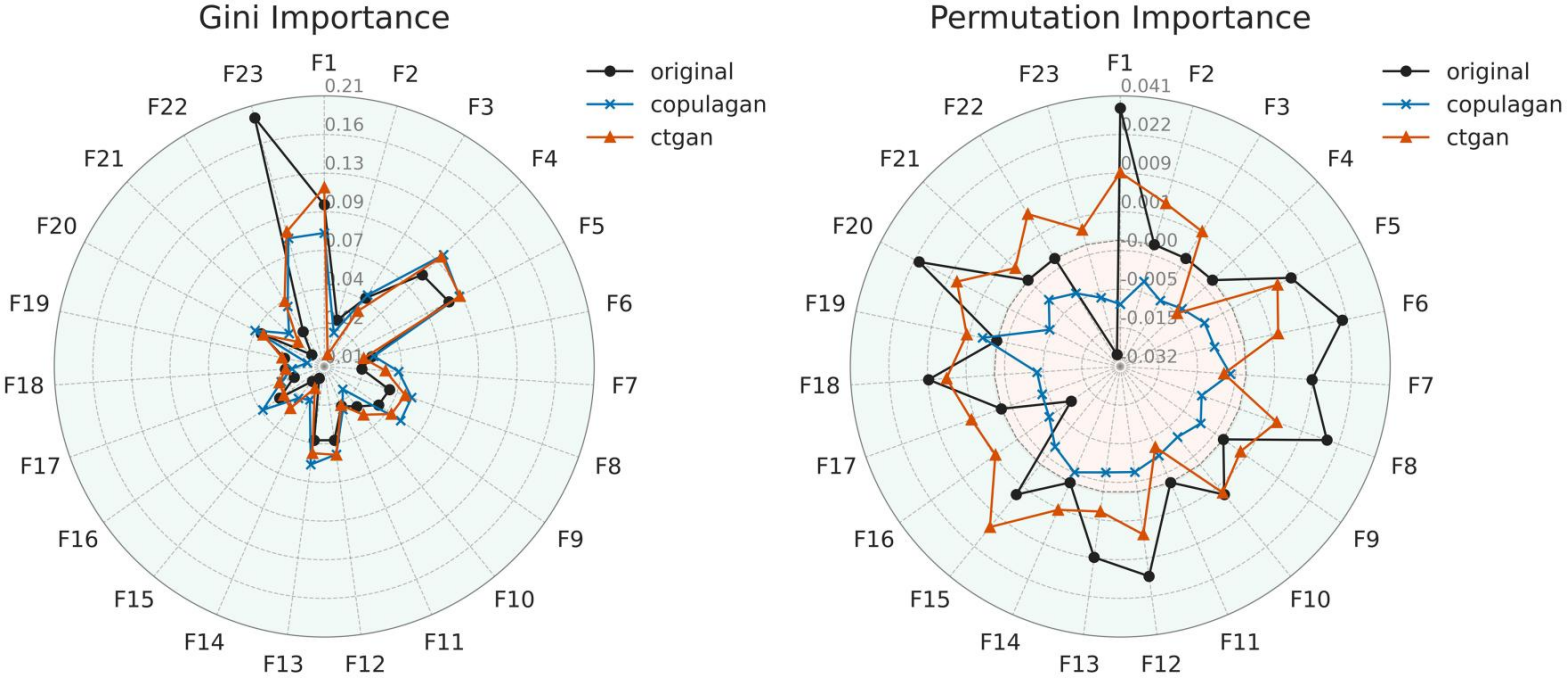
Radar plots of feature importance (Gini and permutation) for the original dataset and 2 synthetic datasets. Feature importance were computed using a Random Forest classifier. The plots highlight the best-performing method (copulagan) and the worst-performing method (ctgan). Feature importance were square root-transformed to enhance the visibility of low-importance features. Radial axis labels reflect the original (pre-transformation) importance values. The background is shaded green for positive values and red for negative values to aid interpretation.

Figure 4 presents VIMP scores for the original dataset and synthetic datasets generated by ctgan and copulagan. In the original data, feature F23 (corresponding to ATC codes) appears as the most influential predictor, with a Gini importance of 0.193. In contrast, both synthetic datasets substantially downweight its relevance, assigning importance scores of 0.077 (copulagan) and 0.082 (ctgan)—a reduction of over 0.11 points. Notably, permutation-based VIMP suggests that the Gini importance may overestimate F23’s contribution in the original data, implying that the synthetic models might provide a more balanced assessment. Both generative approaches appear to correctly identify that F23 performs no better than a random permutation.

In contrast, both models tend to overestimate the Gini importance of less informative features such as F4 and F5, inflating their predictive value relative to the original data. Overall, ctgan more closely reproduces both Gini and permutation-based VIMP scores from the original dataset, indicating that copulagan’s generally strong performance does not extend to accurately preserving individual feature relevance. This observation is supported by supplementary analyses of linkage disequilibrium and univariate distributions, which show higher fidelity in ctgan. The superior overall performance of copulagan likely stems from its ability to better approximate the joint distribution, despite exhibiting certain distributional shifts. This discrepancy highlights an important insight: alignment with univariate or marginal importance metrics does not necessarily translate to improved model utility, particularly when predictive performance depends on complex, higher-order interactions. In other words, there may be a trade-off between matching statistical distributions and optimizing for downstream predictive tasks.

## Discussion

### Principal results

Our findings confirm that SDG methods reveal a fundamental trade-off between privacy risk, measured via ε-identifiability, and broad utility, quantified by pMSE. Deep learning-based models—particularly LLM and tvae trained for 10000 epochs—tend to achieve lower pMSE values, reflecting high fidelity to the marginal and joint distributions of the original data. However, this increased resemblance often comes at the cost of elevated ε-identifiability, suggesting a greater likelihood of re-identification and, hence, a potential compromise of data privacy. In contrast, traditional methods such as copula, synthpop, and avatar typically exhibit lower ε-identifiability while achieving moderate levels of broad utility. Although these models may not attain the same distributional precision as their deep learning counterparts, their ability to preserve privacy while producing usable synthetic data makes them highly relevant in sensitive domains such as PGx, where data protection is paramount.

Specific utility—as measured by the weighted *F*_1_ score ($F_{1}^{w}$)—is not strongly correlated with broad utility. Models achieving low pMSE scores do not necessarily perform better in downstream predictive tasks. For instance, LLM at 10 000 epochs exhibits superior pMSE performance in the phenotype dataset but offers only baseline-level predictive accuracy. Conversely, models like ctgan and copulagan at moderate training durations achieve competitive $F_{1}^{w}$ scores while maintaining acceptable privacy levels.

Despite the growing emphasis on increasingly complex generative models, traditional approaches such as copula and synthpop demonstrate robust performance across both broad (pMSE) and specific ($F_{1}^{w}$) utility metrics, particularly in the high-dimensional genotype setting. While these methods may not always achieve the lowest pMSE values observed with deep learning models, they consistently exhibit lower ε-identifiability, greater stability, and fewer training dependencies. Although avatar yielded only slightly weaker performance in terms of utility and privacy metrics, it exhibited substantially higher variability across sampling iterations, introducing an additional layer of uncertainty. Given this inconsistency and considering the privacy-utility trade-offs observed in our evaluation, copula and synthpop emerge as more reliable and preferred choices for SDG in PGx settings, where reproducibility, interpretability, and risk minimization are critical.

Overall, our results clearly indicate that no single SDG model universally dominates across all evaluation criteria. The performance of models varies substantially depending on the dataset, metric, and training configuration. Importantly, evaluating only one dimension of utility (eg, pMSE or $F_{1}^{w}$ score) may give an incomplete or misleading picture. For a comprehensive assessment of synthetic data quality—especially in healthcare and PGx applications—both privacy and multiple forms of utility must be jointly considered. Our results support the adoption of a multimetric, application-aware benchmarking strategy to ensure the selection of SDG models that align with specific use-case requirements.

The identifiability risks identified in our evaluation have direct consequences for how synthetic data can be shared. In particular, synthetic datasets with high ε-identifiability—often generated by deep learning models trained for extended durations—may still pose privacy risks despite not containing real individuals’ data. This limits their applicability for public release in open science frameworks. While such data may be appropriate for use within tightly controlled multicenter collaborations, unrestricted dissemination would require rigorous privacy assessments and possibly additional safeguards. Consequently, privacy-preserving SDG models such as copula and synthpop are more suitable for scenarios that demand both utility and low re-identification risk, especially when data sharing is intended beyond institutional boundaries.

### Comparison with prior work

Our results indicate that SDG methods vary in their effectiveness when applied to high-dimensional data with few observations compared to datasets with fewer variables and a larger number of observations. In numerous studies, synthpop and ctgan have demonstrated strong performance.^32^^,^^33^ While synthpop and copula are among the most effective methods, ctgan struggles, particularly with high-dimensional data. For instance, synthpop employs techniques such as multivariate imputation to preserve relationships between variables while maintaining the overall data structure. In contrast, ctgan can face significant challenges when dealing with high-dimensional datasets with a limited number of samples. This difficulty stems from the training process of GANs, which requires large amounts of training data to achieve stable and high-quality outcomes. In high-dimensional datasets with few data points, mode collapse and overfitting are likely, undermining the model’s ability to capture the underlying data distributions accurately. As a result, ctgan is particularly prone to performance degradation in such scenarios.

### Limitations

Our study, while comprehensive in exploring different SDG methods, has some specific limitations that should be noted. First, the evaluation of models was constrained by the selection of datasets, which may not fully represent the diversity and complexity found in other real-world data scenarios. For instance, while the phenotype and genotype datasets provide useful insights, they may not capture all nuances of high-dimensional data distributions, potentially limiting the generalizability of our findings to other types of data with different variable structures or distributions. Additionally, while deep learning-based methods like ctgan and tvae were assessed for various epoch sizes, the computational cost of training larger models and the resource-intensive nature of these approaches could pose practical challenges in real-world applications. Specifically, models like synthpop and tabula also required extensive processing time—running for several days on high-performance computing systems—due to the following reasons: synthpop’s CART-based approach became computationally intensive when handling high-cardinality categorical variables, while tabula’s LLM-based architecture inherently demands significant computational resources. This limitation can also affect the reproducibility of results when working with very large datasets or complex model architectures.

Another key limitation is related to the evaluation metrics used to assess data quality and privacy. While metrics such as $F_{1}^{w}$ score, pMSE, and ε-identifiability provide valuable insights into synthetic data utility and privacy risks, they each have inherent shortcomings. For instance, ε-identifiability focuses on re-identification risk but may not fully capture all potential privacy concerns, particularly when considering complex adversarial attacks or data linkage scenarios that are more difficult to measure. Similarly, while the $F_{1}$ score is effective for measuring specific utility, it may not always reflect the preservation of nuanced feature relationships, which is critical for applications that rely on complex data interactions. This indicates that the combination of metrics we employed may not provide a complete picture of the trade-offs between data utility and privacy across all types of SDG methods.

## Conclusions

In conclusion, our study shows that SDG involves a clear trade-off between utility and privacy. Deep learning models with high epoch size may offer high distributional fidelity but often increase re-identification risk. In contrast, simpler methods like copula and synthpop achieve a better privacy-utility balance, making them strong candidates for sensitive domains like PGx. We also find that low pMSE does not guarantee predictive utility, emphasizing the need for multimetric evaluation. No model excels across all criteria, so method selection must align with the specific demands of the application. Synthetic data holds promise for PGx, but success depends on choosing models that balance accuracy, privacy, and stability within real-world constraints.

## Supplementary Material

ooaf107_Supplementary_Data

## Data Availability

The data sets generated and analyzed during this study are not publicly available due to privacy or ethical restrictions but are available on reasonable request from SA.
