# Supplementary material for: Synthetic data for pharmacogenetics: enabling scalable and secure research
Source: JAMIA Open. 2025 Oct 3;8(5):ooaf107. doi: 10.1093/jamiaopen/ooaf107 (PMC12492482; doi:10.1093/jamiaopen/ooaf107)
Supplement: ooaf107_Supplementary_Data [file ooaf107_supplementary_data.zip › Supplementary Material S1.pdf]

Supplementary Material S1: Tables for Linkage Disequilibrium (LD) and Phenotypic Assosciation Matrix

As a representative case study, we compared the linkage disequilibrium (LD) within the CYP2D6 locus between synthetic and original genotype data. The analysis includes the following pharmacogenetically relevant SNPs: rs35742686_CYP2D6_.3_delA, rs3892097_CYP2D6_.4_G>A, rs5030655_CYP2D6_.6_delT, rs5030656_CYP2D6_.9_delAAG, rs1065852_CYP2D6_.4.10_C>T, rs59421388_CYP2D6_.29_G>A, and rs28371725_CYP2D6_.41_G>A. These variants span functionally critical regions of the CYP2D6 gene and represent key haplotypes involved in drug metabolism. Pairwise LD was quantified using standard metrics (D, D', and r^2) to assess local haplotype architecture and inform downstream genotype-to-phenotype modeling.

Similarly, we assessed the phenotype dataset by comparing the Phenotypic Association Matrix derived from pharmacogene-inferred metabolizer statuses (poor, intermediate, normal, and ultra-rapid) across synthetic and original data. This comparison enables the evaluation of genotype–phenotype structure retention in synthetic datasets.

Linkage Disequilibrium (LD)

Table S1.1: Comparison of LD structure within the CYP2D6 locus between original and synthetic genotype data. Pairwise LD metrics (D, D', and r^2) were computed across pharmacogenetically relevant SNPs to assess the preservation of haplotype architecture.

Model	SNP A	SNP B	$D_{synthetic}$	$D'_{synthetic}$	$r^2_{synthetic}$	$D_{original}$	$D'_{original}$	$r^2_{original}$	$D_{\|synthetic-original\|}$	$\rho(D'_{synthetic}, D'_{original})$	$r^2_{\|synthetic-original\|}$
copulagan (batch size=50, epochs=300)	**rs1065852_CYP2D6_.4_.10_C>T**	**rs28371725_CYP2D6_.41_G>A**	-0.006	-0.298	0.004	-0.011	-0.860	0.012	0.006	0.35	0.009
	**rs1065852_CYP2D6_.4_.10_C>T**	**rs59421388_CYP2D6_.29_G>A**	0.000	-0.658	0.002	0.001	0.420	0.004	0.002		0.002
	**rs35742686_CYP2D6_.3_delA**	**rs1065852_CYP2D6_.4_.10_C>T**	0.000	-0.360	0.002	-0.004	-1.000	0.005	0.004		0.003
	**rs35742686_CYP2D6_.3_delA**	**rs28371725_CYP2D6_.41_G>A**	0.000	-0.396	0.002	-0.003	-1.000	0.003	0.003		0.002
	**rs35742686_CYP2D6_.3_delA**	**rs3892097_CYP2D6_.4_G>A**	0.000	-0.286	0.002	-0.004	-1.000	0.005	0.004		0.003
	**rs35742686_CYP2D6_.3_delA**	**rs5030655_CYP2D6_.6_delT**	0.000	-0.794	0.002	-0.001	-1.000	0.001	0.001		0.001
	**rs35742686_CYP2D6_.3_delA**	**rs5030656_CYP2D6_.9_delAAG**	0.000	-0.690	0.002	0.004	0.144	0.012	0.004		0.009
	**rs35742686_CYP2D6_.3_delA**	**rs59421388_CYP2D6_.29_G>A**	0.000	-0.884	0.004	0.000	-1.000	0.000	0.000		0.003
	**rs3892097_CYP2D6_.4_G>A**	**rs1065852_CYP2D6_.4_.10_C>T**	0.021	0.150	0.021	0.065	0.581	0.317	0.045		0.296
	**rs3892097_CYP2D6_.4_G>A**	**rs28371725_CYP2D6_.41_G>A**	-0.005	-0.255	0.003	-0.011	-0.853	0.011	0.006		0.008
	**rs3892097_CYP2D6_.4_G>A**	**rs5030655_CYP2D6_.6_delT**	-0.001	-0.409	0.002	-0.004	-1.000	0.005	0.003		0.003
	**rs3892097_CYP2D6_.4_G>A**	**rs5030656_CYP2D6_.9_delAAG**	-0.003	-0.488	0.003	-0.007	-1.000	0.008	0.004		0.005
	**rs3892097_CYP2D6_.4_G>A**	**rs59421388_CYP2D6_.29_G>A**	0.000	-0.472	0.002	0.001	0.425	0.004	0.001		0.003
	**rs5030655_CYP2D6_.6_delT**	**rs1065852_CYP2D6_.4_.10_C>T**	-0.001	-0.405	0.002	-0.004	-1.000	0.005	0.003		0.003
	**rs5030655_CYP2D6_.6_delT**	**rs28371725_CYP2D6_.41_G>A**	0.000	-0.227	0.001	0.001	0.034	0.000	0.001		0.001
	**rs5030655_CYP2D6_.6_delT**	**rs5030656_CYP2D6_.9_delAAG**	0.001	-0.329	0.005	0.000	0.012	0.000	0.001		0.005
	**rs5030655_CYP2D6_.6_delT**	**rs59421388_CYP2D6_.29_G>A**	0.000	-0.574	0.009	0.000	-1.000	0.000	0.000		0.009
	**rs5030656_CYP2D6_.9_delAAG**	**rs1065852_CYP2D6_.4_.10_C>T**	-0.003	-0.636	0.003	-0.007	-1.000	0.009	0.004		0.005
	**rs5030656_CYP2D6_.9_delAAG**	**rs28371725_CYP2D6_.41_G>A**	0.003	0.061	0.005	0.003	0.054	0.002	0.001		0.004
	**rs5030656_CYP2D6_.9_delAAG**	**rs59421388_CYP2D6_.29_G>A**	0.000	-0.627	0.006	0.000	-1.000	0.000	0.000		0.006
	**rs59421388_CYP2D6_.29_G>A**	**rs28371725_CYP2D6_.41_G>A**	0.000	-0.633	0.002	0.000	-1.000	0.000	0.000		0.001
ctgan (batch size=50, epochs=10000)	**rs1065852_CYP2D6_.4_.10_C>T**	**rs28371725_CYP2D6_.41_G>A**	-0.005	-0.741	0.005	-0.011	-0.860	0.012	0.006	0.49	0.007
	**rs1065852_CYP2D6_.4_.10_C>T**	**rs59421388_CYP2D6_.29_G>A**	0.000	-0.510	0.002	0.001	0.420	0.004	0.001		0.002
	**rs35742686_CYP2D6_.3_delA**	**rs1065852_CYP2D6_.4_.10_C>T**	0.000	-0.397	0.002	-0.004	-1.000	0.005	0.004		0.002
	**rs35742686_CYP2D6_.3_delA**	**rs28371725_CYP2D6_.41_G>A**	0.000	-0.632	0.003	-0.003	-1.000	0.003	0.003		0.001
	**rs35742686_CYP2D6_.3_delA**	**rs3892097_CYP2D6_.4_G>A**	0.000	-0.414	0.003	-0.004	-1.000	0.005	0.004		0.002
	**rs35742686_CYP2D6_.3_delA**	**rs5030655_CYP2D6_.6_delT**	0.000	-0.838	0.001	-0.001	-1.000	0.001	0.001		0.001
	**rs35742686_CYP2D6_.3_delA**	**rs5030656_CYP2D6_.9_delAAG**	0.000	-0.529	0.004	0.004	0.144	0.012	0.004		0.008
	**rs35742686_CYP2D6_.3_delA**	**rs59421388_CYP2D6_.29_G>A**	0.000	-0.896	0.003	0.000	-1.000	0.000	0.000		0.003
	**rs3892097_CYP2D6_.4_G>A**	**rs1065852_CYP2D6_.4_.10_C>T**	0.017	0.170	0.026	0.065	0.581	0.317	0.048		0.292
	**rs3892097_CYP2D6_.4_G>A**	**rs28371725_CYP2D6_.41_G>A**	-0.005	-0.622	0.005	-0.011	-0.853	0.011	0.005		0.006
	**rs3892097_CYP2D6_.4_G>A**	**rs5030655_CYP2D6_.6_delT**	0.000	-0.291	0.002	-0.004	-1.000	0.005	0.004		0.002
	**rs3892097_CYP2D6_.4_G>A**	**rs5030656_CYP2D6_.9_delAAG**	-0.004	-0.573	0.004	-0.007	-1.000	0.008	0.003		0.004
	**rs3892097_CYP2D6_.4_G>A**	**rs59421388_CYP2D6_.29_G>A**	0.001	-0.292	0.003	0.001	0.425	0.004	0.001		0.001
	**rs5030655_CYP2D6_.6_delT**	**rs1065852_CYP2D6_.4_.10_C>T**	0.000	-0.330	0.002	-0.004	-1.000	0.005	0.004		0.003
	**rs5030655_CYP2D6_.6_delT**	**rs28371725_CYP2D6_.41_G>A**	0.000	-0.463	0.002	0.001	0.034	0.000	0.001		0.001
	**rs5030655_CYP2D6_.6_delT**	**rs5030656_CYP2D6_.9_delAAG**	0.000	-0.317	0.002	0.000	0.012	0.000	0.000		0.002
	**rs5030655_CYP2D6_.6_delT**	**rs59421388_CYP2D6_.29_G>A**	0.000	-0.862	0.002	0.000	-1.000	0.000	0.000		0.002
	**rs5030656_CYP2D6_.9_delAAG**	**rs1065852_CYP2D6_.4_.10_C>T**	-0.003	-0.623	0.004	-0.007	-1.000	0.009	0.004		0.005
	**rs5030656_CYP2D6_.9_delAAG**	**rs28371725_CYP2D6_.41_G>A**	0.003	0.020	0.008	0.003	0.054	0.002	0.001		0.006
	**rs5030656_CYP2D6_.9_delAAG**	**rs59421388_CYP2D6_.29_G>A**	0.000	-0.531	0.003	0.000	-1.000	0.000	0.001		0.003
	**rs59421388_CYP2D6_.29_G>A**	**rs28371725_CYP2D6_.41_G>A**	0.000	-0.620	0.002	0.000	-1.000	0.000	0.000		0.002

Phenotypic Association Matrix

Table S1.2: Comparison of Phenotypic Association Matrices derived from pharmacogene-inferred metabolizer statuses (PM, IM, NM, UM) in original and synthetic phenotype datasets, highlighting the degree to which genotype–phenotype associations are retained.

Model	Gene A	Gene B	$D_{synthetic}$	$D'_{synthetic}$	$r^2_{synthetic}$	$D_{original}$	$D'_{original}$	$r^2_{original}$	$D_{\|synthetic-original\|}$	$\rho(D'_{synthetic}, D'_{original})$	$r^2_{\|synthetic-original\|}$
	**CYP2B6**	**CYP2C9**	0.02192	0.22923	0.00688	0.03795	0.38770	0.01268	0.01603		0.00580
	**CYP2B6**	**CYP2D6**	0.02371	0.40177	0.00889	0.03422	0.56468	0.01347	0.01051		0.00458
	**CYP2B6**	**CYP3A5**	0.01697	0.33762	0.00837	0.01746	0.29120	0.00580	0.00050		0.00257
	**CYP2B6**	**DPYD**	0.01661	0.29974	0.00706	0.04174	0.83649	0.04366	0.02513		0.03660
	**CYP2B6**	**TPMT**	0.00938	0.45905	0.00782	0.02276	0.35726	0.02336	0.01338		0.01554
	**CYP2C19**	**CYP2B6**	0.02154	0.32894	0.00809	0.02606	0.34735	0.00803	0.00452		0.00006
	**CYP2C19**	**CYP2C9**	0.02250	0.32815	0.00733	0.03082	0.55222	0.01186	0.00832		0.00453
	**CYP2C19**	**CYP2D6**	0.02031	0.47446	0.00722	0.02768	0.69211	0.00980	0.00737		0.00259
	**CYP2C19**	**CYP3A5**	0.01581	0.40741	0.00741	0.01211	0.41891	0.00399	0.00370		0.00342
	**CYP2C19**	**DPYD**	0.01691	0.38033	0.00783	0.01254	0.61022	0.00444	0.00437		0.00338
**copulagan (batch size=50, epochs=300)**	**CYP2C19**	**TPMT**	0.00961	0.49322	0.00778	0.01303	0.63134	0.00830	0.00342	0.39	0.00052
	**CYP2C9**	**CYP2D6**	0.02234	0.37752	0.00726	0.02941	0.56897	0.00911	0.00707		0.00185
	**CYP2C9**	**CYP3A5**	0.01699	0.31645	0.00797	0.03114	0.31777	0.01778	0.01415		0.00982
	**CYP2C9**	**DPYD**	0.01745	0.26345	0.00726	0.02173	0.49761	0.01294	0.00428		0.00568
	**CYP2C9**	**TPMT**	0.00982	0.43548	0.00817	0.03795	1.00000	0.07359	0.02814		0.06542
	**CYP2D6**	**CYP3A5**	0.01586	0.48186	0.00730	0.02390	0.64599	0.01034	0.00804		0.00303
	**CYP2D6**	**DPYD**	0.01704	0.45865	0.00748	0.03439	0.76507	0.02738	0.01734		0.01990
	**CYP2D6**	**TPMT**	0.00983	0.60831	0.00874	0.00243	0.52961	0.00043	0.00739		0.00831
	**CYP3A5**	**DPYD**	0.01271	0.36372	0.00734	0.01930	0.66608	0.01457	0.00659		0.00723
	**CYP3A5**	**TPMT**	0.00750	0.59713	0.00932	0.01168	1.00000	0.01154	0.00418		0.00222
	**DPYD**	**TPMT**	0.00616	0.44280	0.00688	0.01233	0.47666	0.01797	0.00616		0.01109
	**CYP2B6**	**CYP2C9**	0.04759	0.31374	0.02818	0.03795	0.38770	0.01268	0.00963		0.01550
	**CYP2B6**	**CYP2D6**	0.03460	0.39443	0.01680	0.03422	0.56468	0.01347	0.00038		0.00333
	**CYP2B6**	**CYP3A5**	0.02189	0.41077	0.01349	0.01746	0.29120	0.00580	0.00443		0.00769
	**CYP2B6**	**DPYD**	0.02292	0.46226	0.01425	0.04174	0.83649	0.04366	0.01881		0.02941
	**CYP2B6**	**TPMT**	0.02166	0.31694	0.02177	0.02276	0.35726	0.02336	0.00110		0.00159
	**CYP2C19**	**CYP2B6**	0.03345	0.36322	0.01495	0.02606	0.34735	0.00803	0.00739		0.00692
	**CYP2C19**	**CYP2C9**	0.03566	0.32874	0.01330	0.03082	0.55222	0.01186	0.00484		0.00145
	**CYP2C19**	**CYP2D6**	0.03158	0.51451	0.01482	0.02768	0.69211	0.00980	0.00390		0.00502
	**CYP2C19**	**CYP3A5**	0.03059	0.40840	0.02041	0.01211	0.41891	0.00399	0.01848		0.01642
	**CYP2C19**	**DPYD**	0.01985	0.50645	0.01067	0.01254	0.61022	0.00444	0.00730		0.00622
**ctgan (batch size=50, epochs=10000))**	**CYP2C19**	**TPMT**	0.01343	0.47286	0.00846	0.01303	0.63134	0.00830	0.00040	0.11	0.00015
	**CYP2C9**	**CYP2D6**	0.05267	0.41803	0.03232	0.02941	0.56897	0.00911	0.02326		0.02321
	**CYP2C9**	**CYP3A5**	0.03027	0.39034	0.01917	0.03114	0.31777	0.01778	0.00087		0.00138
	**CYP2C9**	**DPYD**	0.02946	0.35325	0.01986	0.02173	0.49761	0.01294	0.00773		0.00692
	**CYP2C9**	**TPMT**	0.03645	0.59693	0.05344	0.03795	1.00000	0.07359	0.00151		0.02015
	**CYP2D6**	**CYP3A5**	0.01487	0.55618	0.00707	0.02390	0.64599	0.01034	0.00902		0.00326
	**CYP2D6**	**DPYD**	0.02090	0.48744	0.01586	0.03439	0.76507	0.02738	0.01348		0.01152
	**CYP2D6**	**TPMT**	0.02710	0.53024	0.04010	0.00243	0.52961	0.00043	0.02467		0.03966
	**CYP3A5**	**DPYD**	0.02003	0.75223	0.01794	0.01930	0.66608	0.01457	0.00073		0.00337
	**CYP3A5**	**TPMT**	0.01163	0.83319	0.01067	0.01168	1.00000	0.01154	0.00005		0.00087
	**DPYD**	**TPMT**	0.00810	0.39370	0.00946	0.01233	0.47666	0.01797	0.00423		0.00851
