# Supplementary material for: Synthetic data for pharmacogenetics: enabling scalable and secure research
Source: JAMIA Open. 2025 Oct 3;8(5):ooaf107. doi: 10.1093/jamiaopen/ooaf107 (PMC12492482; doi:10.1093/jamiaopen/ooaf107)
Supplement: ooaf107_Supplementary_Data [file ooaf107_supplementary_data.zip › Supplementary Material S2.pdf]

Supplementary Material S2: Univariate Plots for selected variables

Comparison of relative frequencies of gene-based metabolizer phenotypes between original data and synthetic data generated by copulagan and ctgan

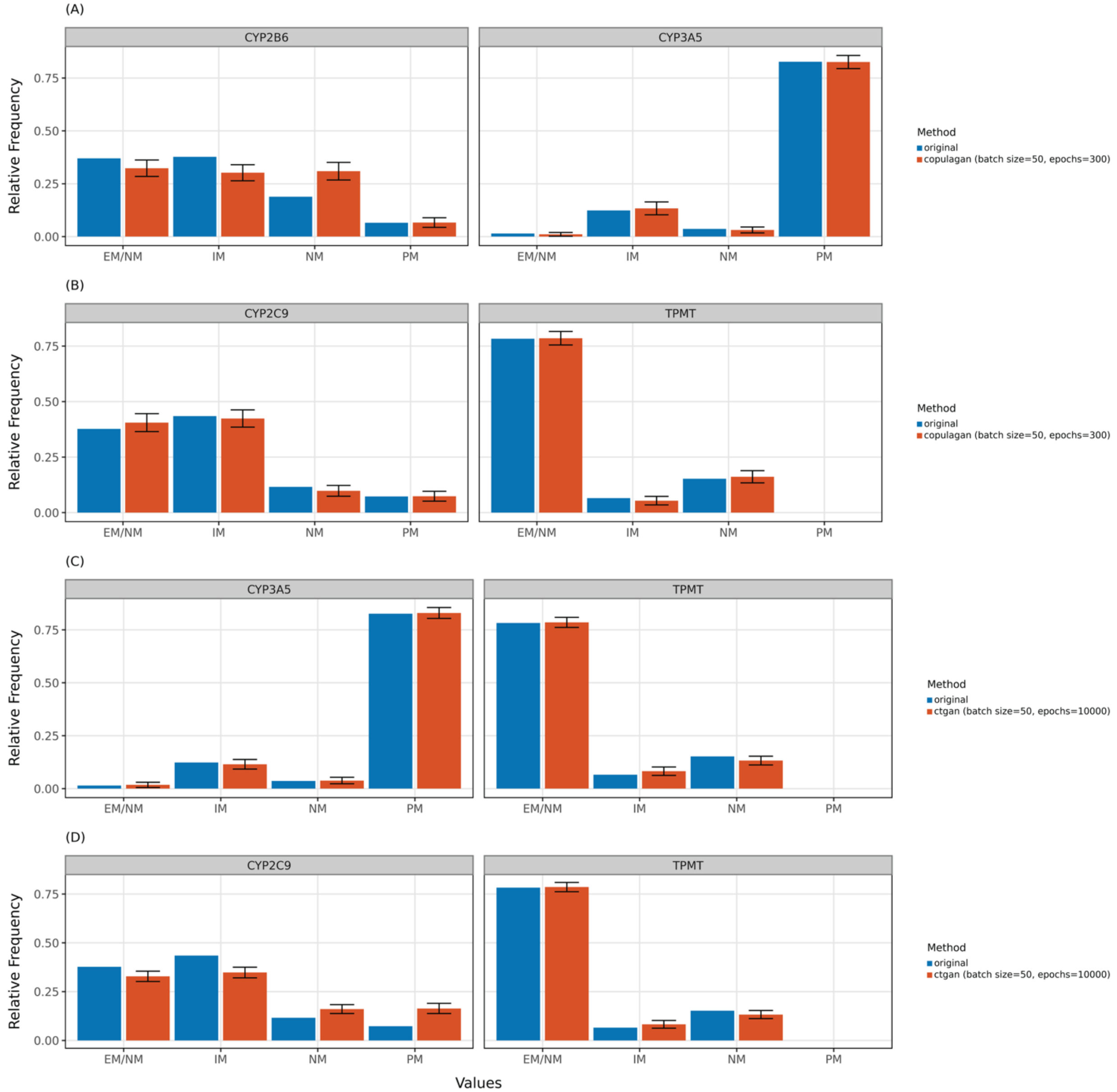

Figure S2.1: Bar plots of categorical phenotype frequencies for selected gene pairs in original data and synthetic data generated by the best (copulagan; batch = 50, epochs = 300) and worst (ctgan; batch = 50, epochs = 10,000) performing SDG methods. Gene pairings follow the grouping in Supplementary Material S2. Panels A-B and C-D display the combinations with the lowest and highest D' differences for each method according to Supplementary Material S2. Error bars represent standard errors across 100 synthetic replications.

Comparison of relative frequencies of SNP values (genotype) between original data and synthetic data generated by copulagan and ctgan

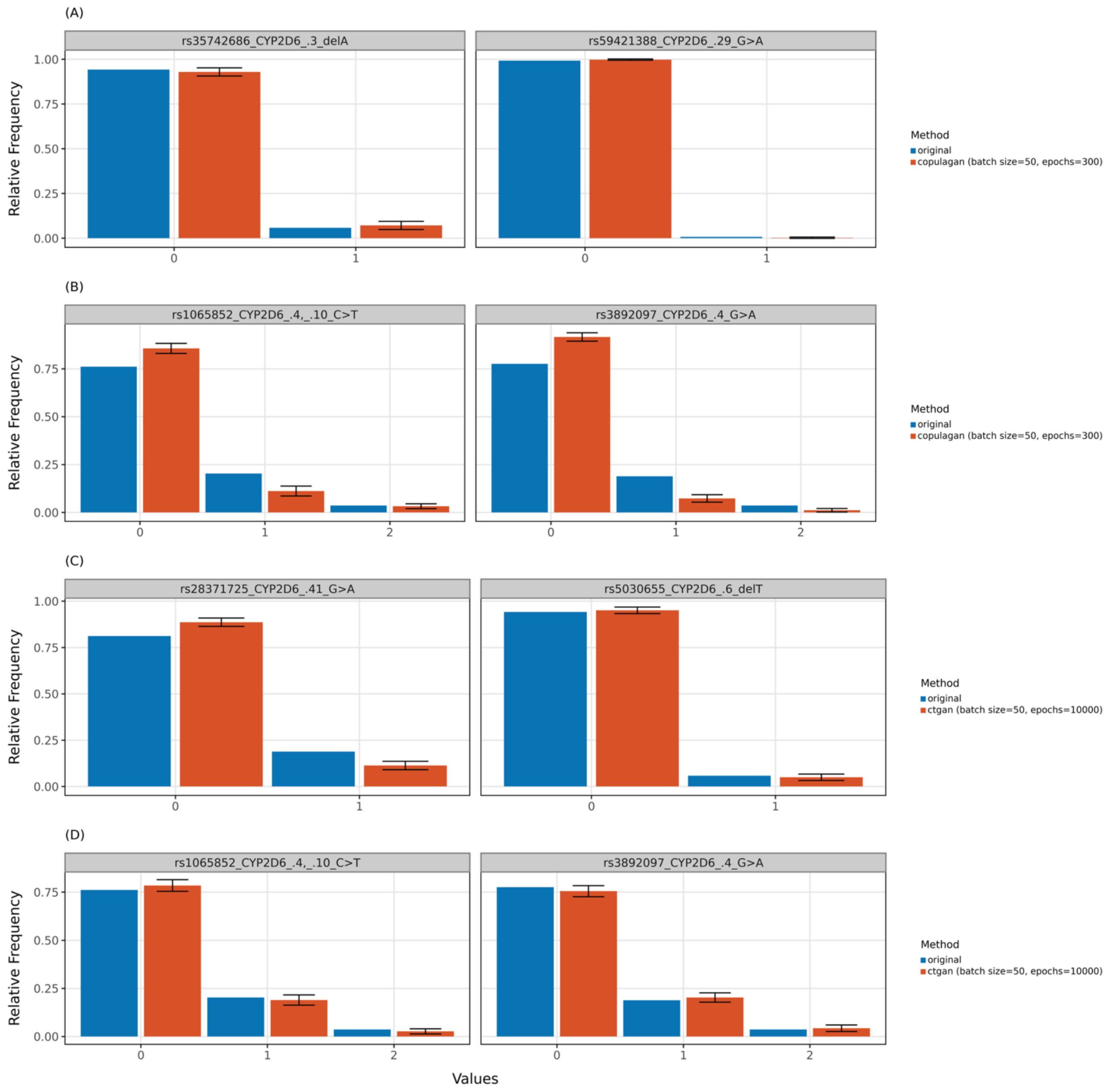

Figure S2.2: Bar plots of SNP value frequencies for SNP pairs in original data and synthetic data generated by the best (copulagan; batch = 50, epochs = 300) and worst (ctgan; batch = 50, epochs = 10,000) performing SDG methods. SNP pairings follow the grouping in Supplementary Material S2. Panels A-B and C-D display the combinations with the lowest and highest D' differences for each method according to Supplementary Material S2. Error bars represent standard errors across 100 synthetic replications.

Comparison of the distribution of the variable "ageRegistration" between original and synthetic datasets generated using copulagan and ctgan for phenotype and genotype data.

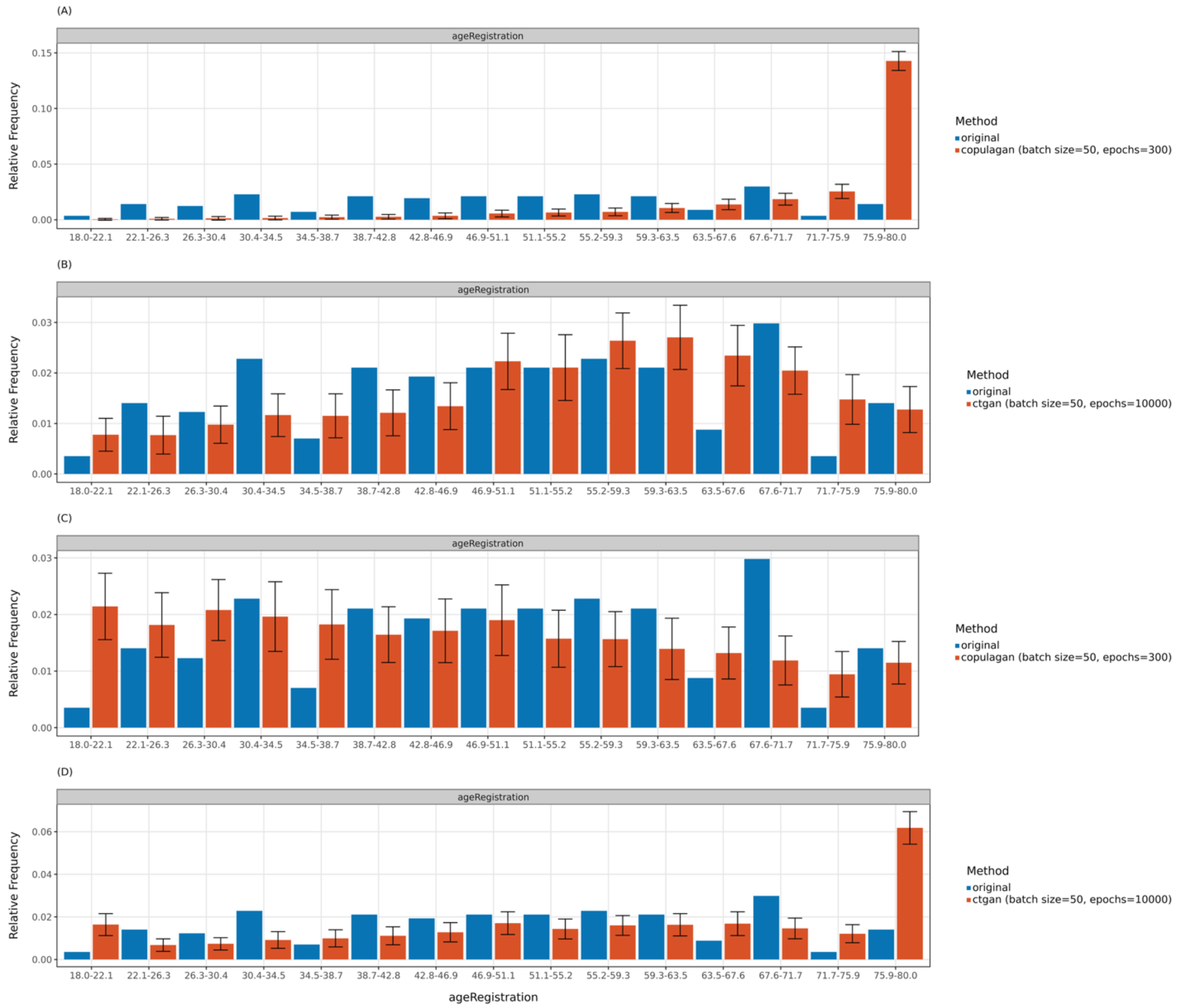

Figure S2.3: Panels (A)–(D) show relative frequency distributions across "ageRegistration" bins for phenotype data (A, B) and genotype data (C, D), generated by the best (copulagan; batch = 50, epochs = 300) and worst (ctgan; batch = 50, epochs = 10,000) performing SDG methods. Blue bars represent original data, orange bars synthetic data. Error bars represent standard errors across 100 synthetic replications.

Comparison of the distribution of the variable “mainDiagnosis” between original and synthetic datasets generated using copulagan and ctgan for phenotype and genotype data.

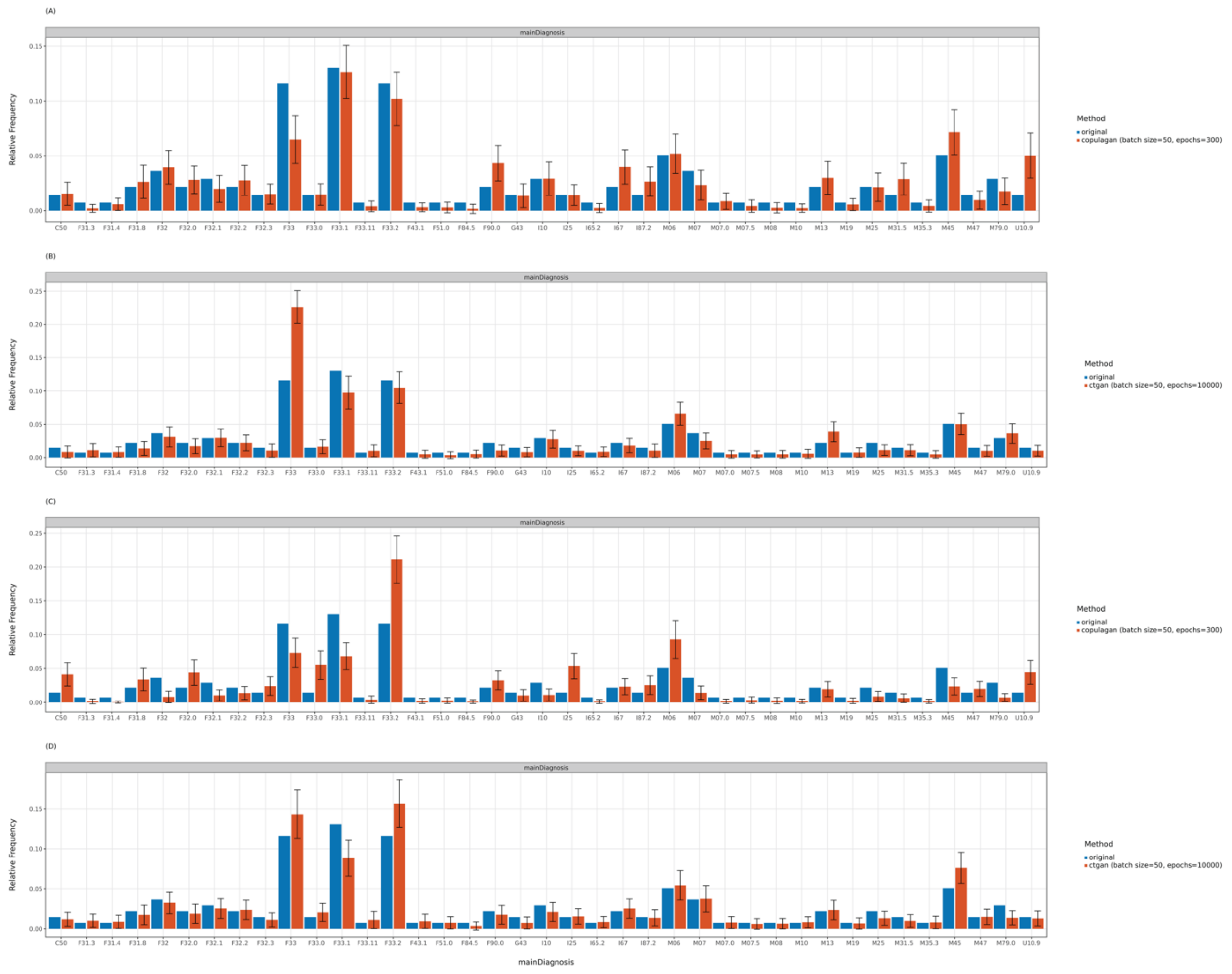

Figure S2.4: Panels (A)–(D) show relative frequency distributions across “mainDiagnosis” bins for phenotype data (A, B) and genotype data (C, D), generated by the best (copulagan; batch = 50, epochs = 300) and worst (ctgan; batch = 50, epochs = 10,000) performing SDG methods. Blue bars represent original data, orange bars synthetic data. Error bars represent standard errors across 100 synthetic replications.

Comparison of the distribution of the variable “changedDrug” between original and synthetic datasets generated using copulagan and ctgan for phenotype and genotype data.

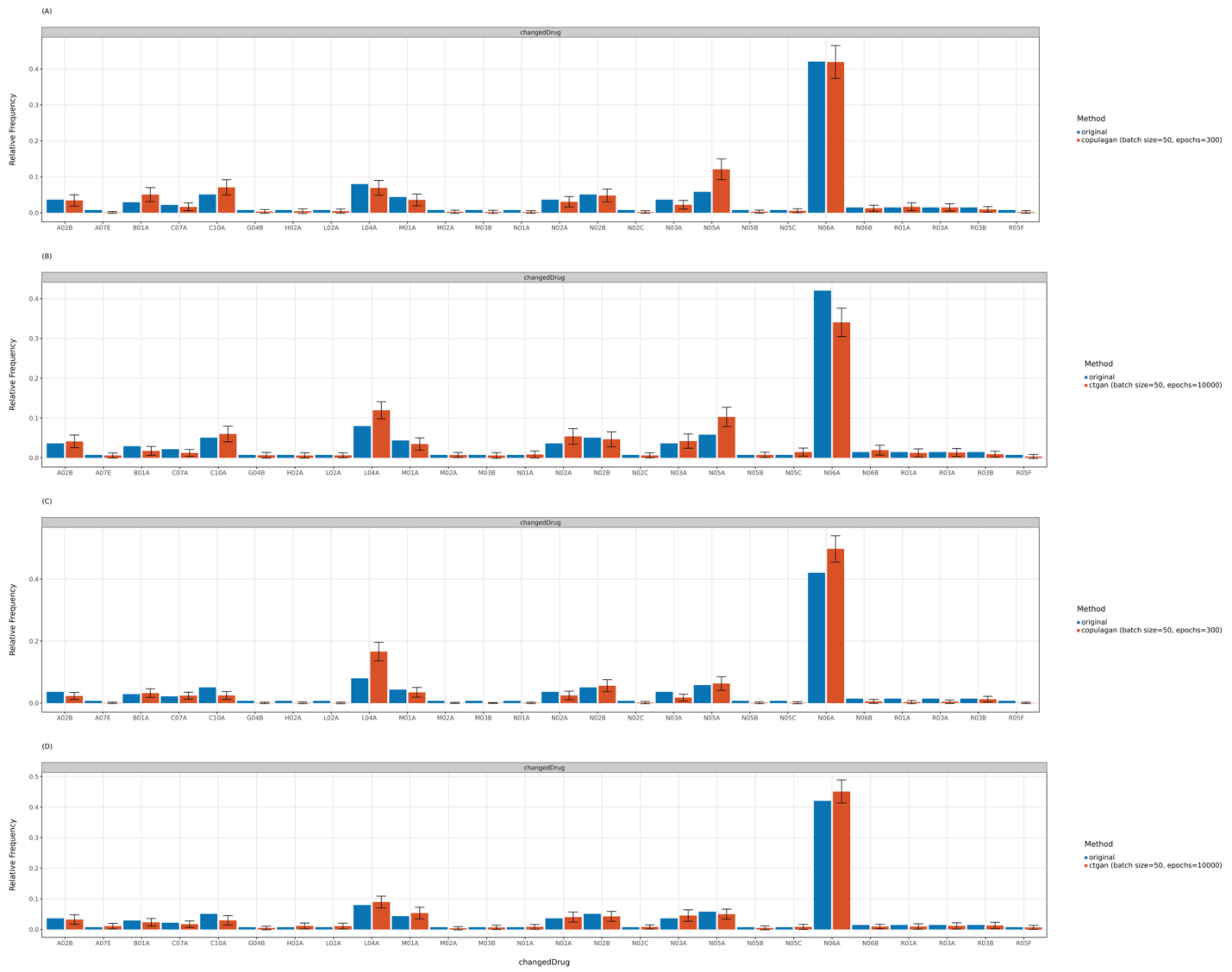

Figure S2.5: Panels (A)–(D) show relative frequency distributions across “changedDrug” bins for phenotype data (A, B) and genotype data (C, D), generated by the best (copulagan; batch = 50, epochs = 300) and worst (ctgan; batch = 50, epochs = 10,000) performing SDG methods. Blue bars represent original data, orange bars synthetic data. Error bars represent standard errors across 100 synthetic replications.
