# Supplementary material for: Synthetic data for pharmacogenetics: enabling scalable and secure research
Source: JAMIA Open. 2025 Oct 3;8(5):ooaf107. doi: 10.1093/jamiaopen/ooaf107 (PMC12492482; doi:10.1093/jamiaopen/ooaf107)
Supplement: ooaf107_Supplementary_Data [file ooaf107_supplementary_data.zip › Supplementary Material S3.pdf]

Supplementary Material S3: Selected Boxplots for SDG Methods on genotype and phenotype data

Variability in ε-Identifiability across SDG methods on the genotype data

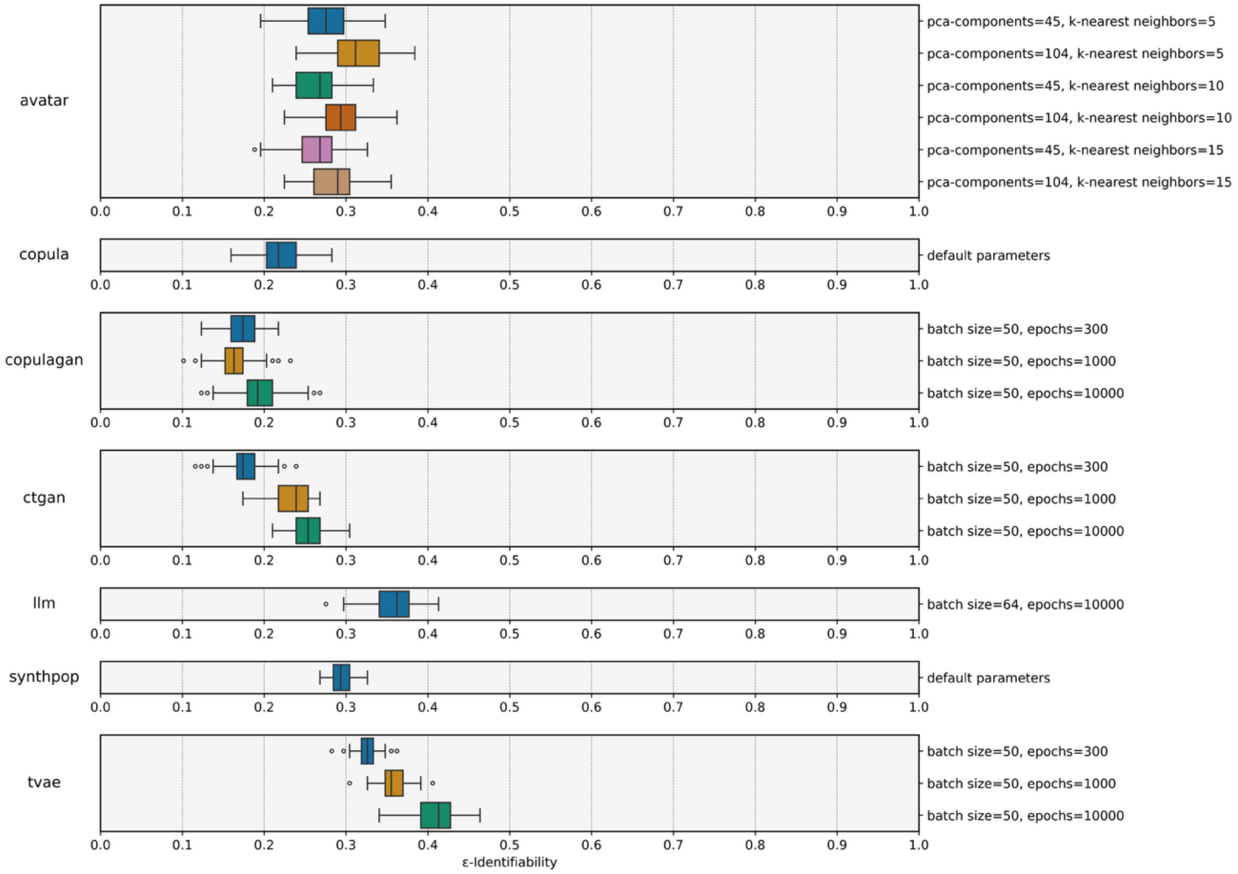

Figure 3.1: Boxplots of the ε-identifiability scores for the genotype data across various generation methods and parameter configurations.

Variability in specific utility (F_1^w) across SDG methods on the genotype data

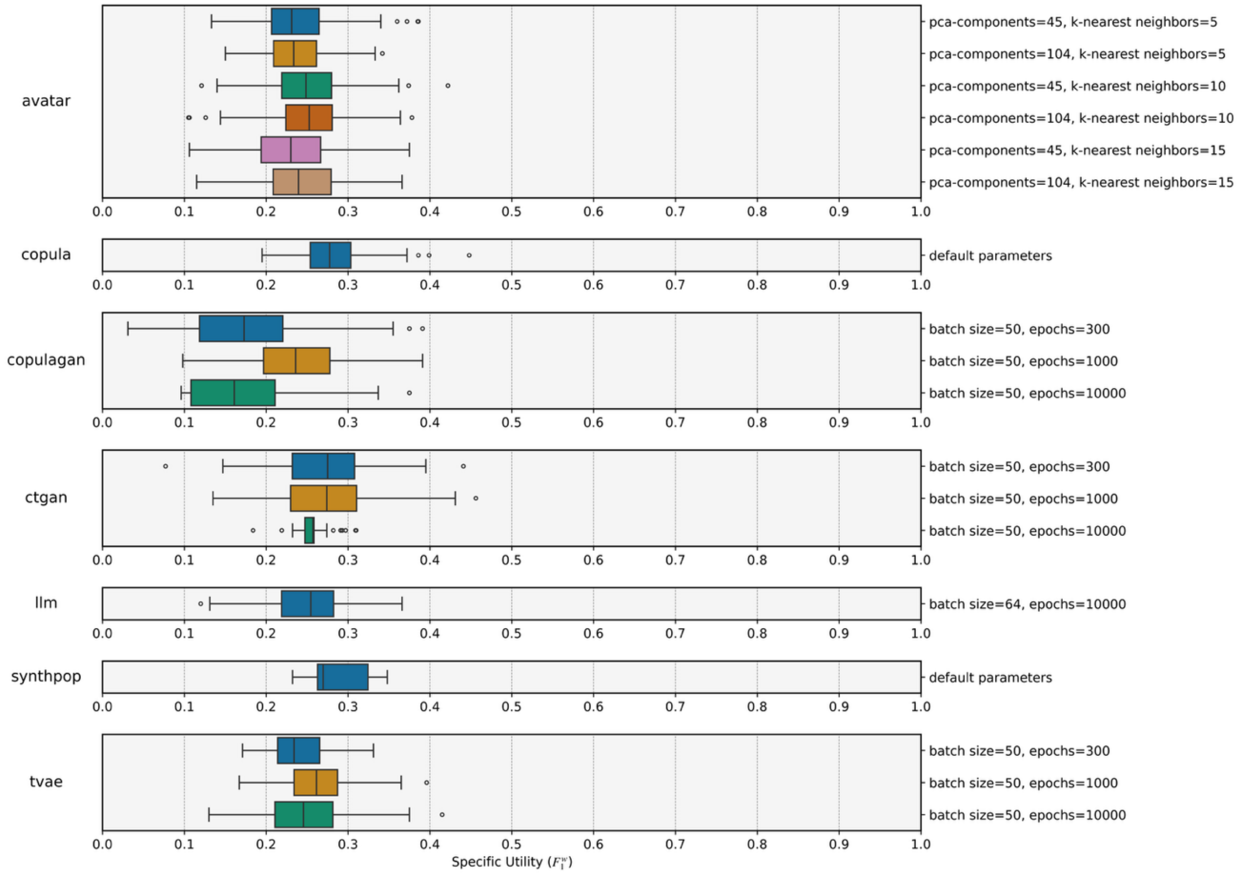

Figure S3.2: Boxplots of the Specific Utility (F_1^w) on the downstream classification task for the genotype data across various generation methods and parameter configurations.

Variability in broad utility ($pMSE$) values across SDG methods on genotype data

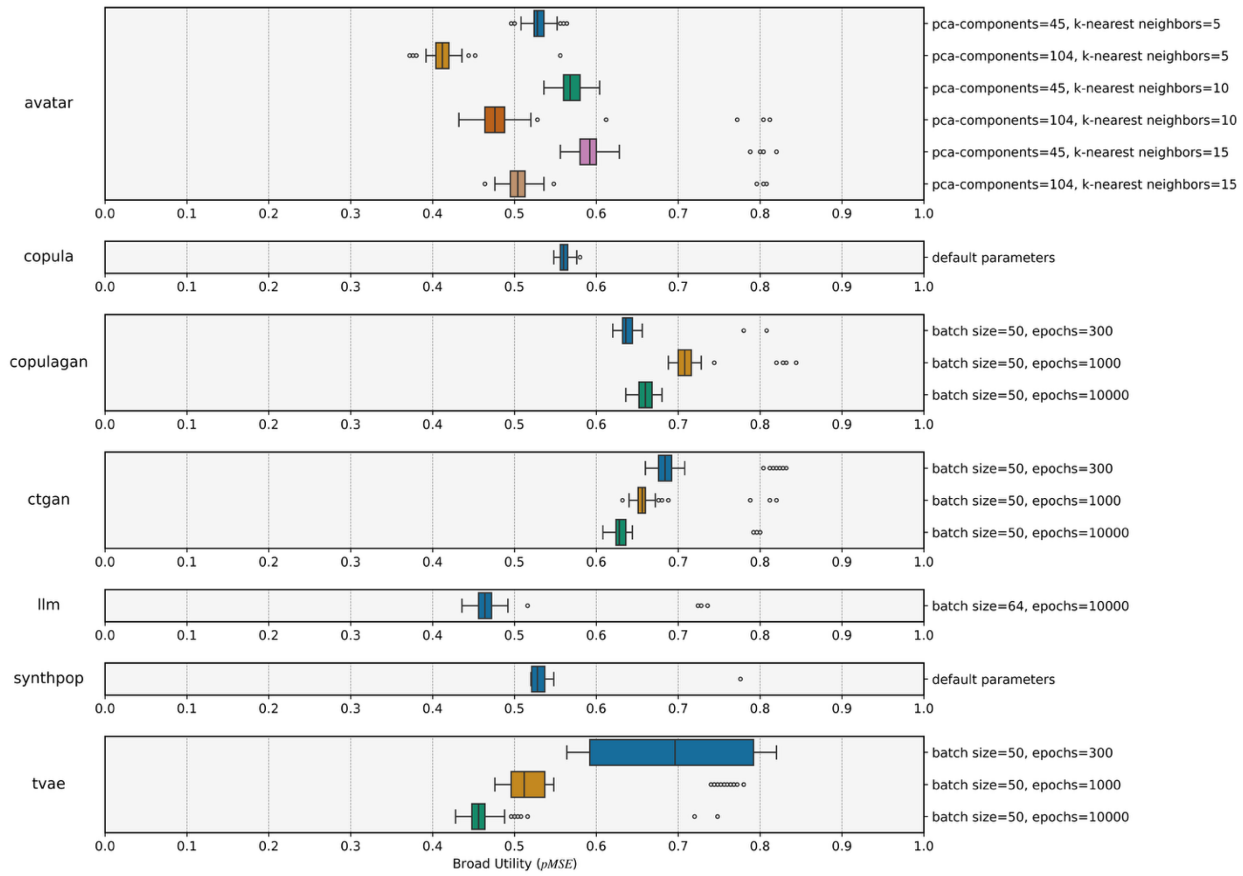

Figure S3.3: Boxplots of the Broad Utility ($pMSE$) values for the genotype data across various generation methods and parameter configurations. Lower Broad Utility ($pMSE$) values indicate higher utility, as ideal synthetic data should be indistinguishable from the original. The metric is normalized such that 0 indicates perfect similarity and 1 reflects maximum distinguishability.

Variability in ε-Identifiability across SDG methods on the phenotype data

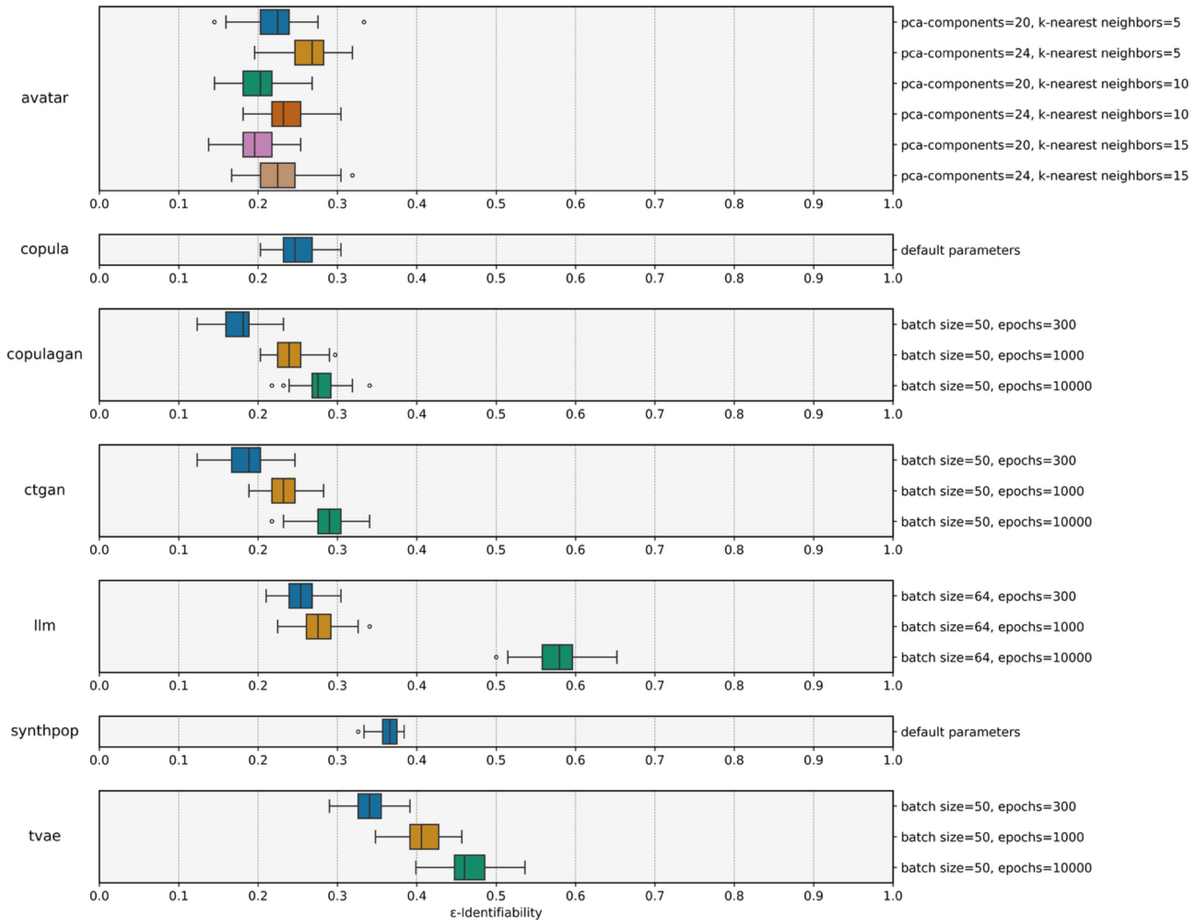

Figure 3.4: Boxplots of the ε-identifiability scores for the phenotype data across various generation methods and parameter configurations.

Variability in specific utility (F_1^w) across SDG methods on the phenotype data

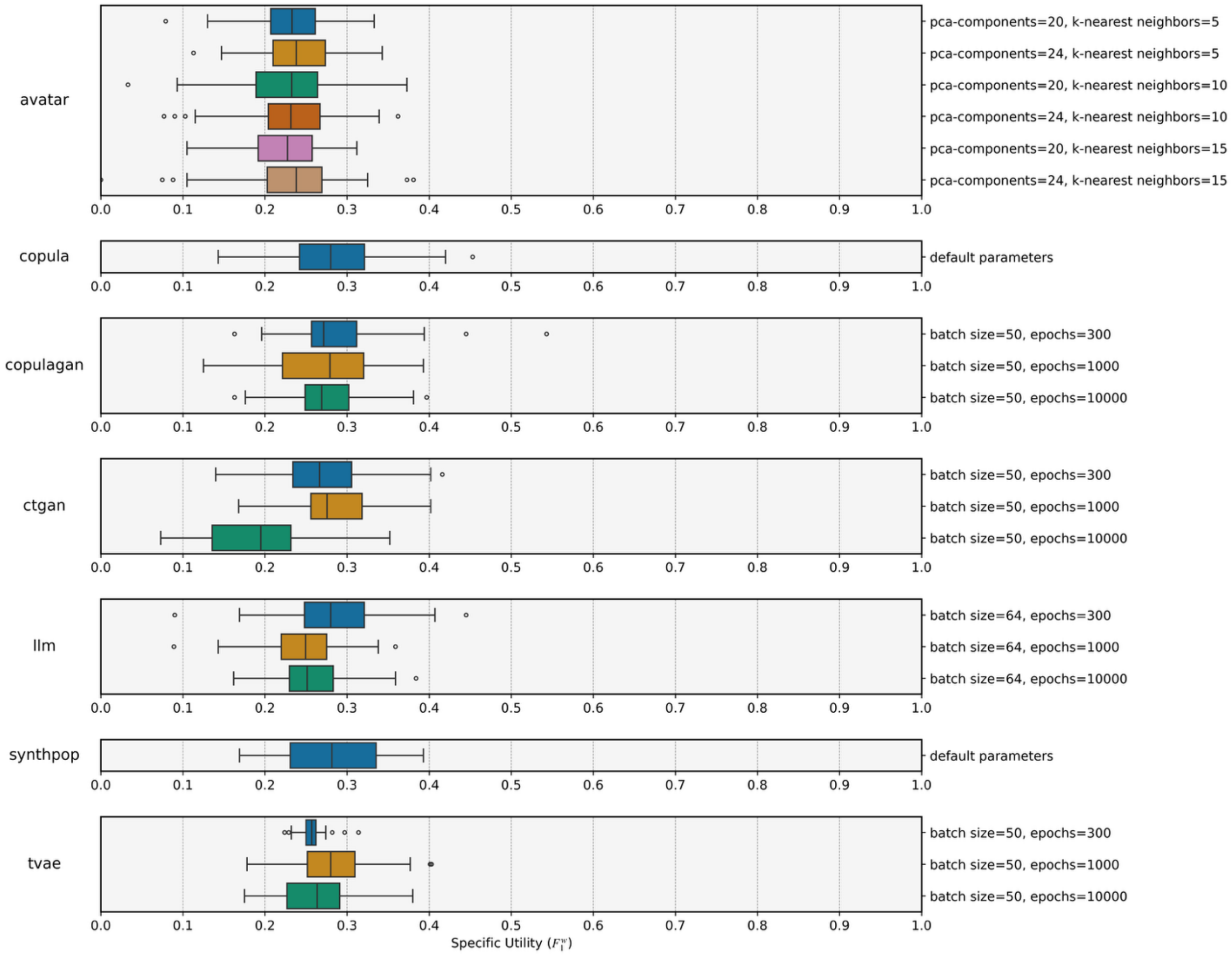

Figure 3.5: Boxplots of the Specific Utility (F_1^w) on the downstream classification task for the phenotype data across various generation methods and parameter configurations.

Variability in broad utility ($pMSE$) values across SDG methods on phenotype data

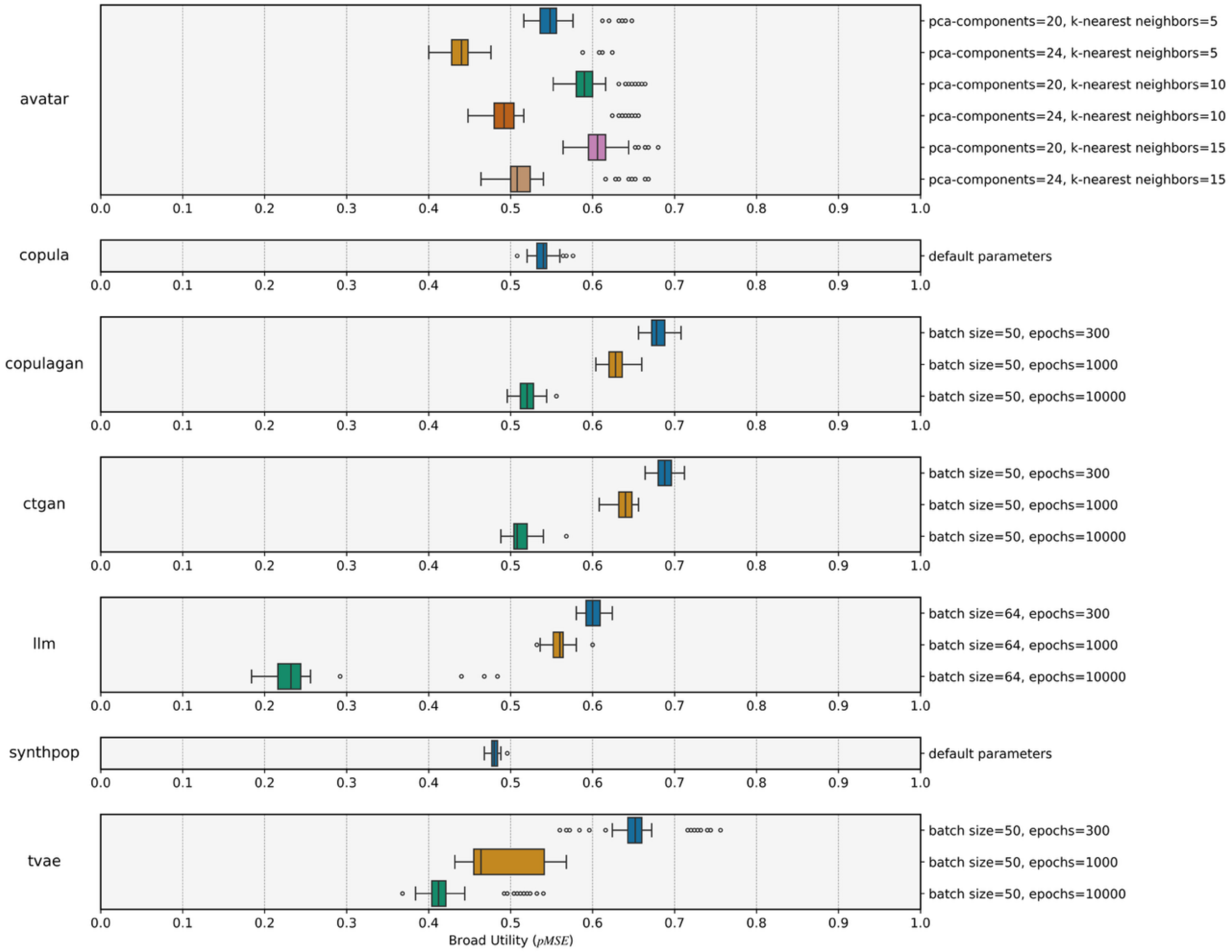

Figure 3.6: Boxplots of the Broad Utility ($pMSE$) values for the phenotype data across various generation methods and parameter configurations. Lower Broad Utility ($pMSE$) values indicate higher utility, as ideal synthetic data should be indistinguishable from the original. The metric is normalized such that 0 indicates perfect similarity and 1 reflects maximum distinguishability.
